# Different predictive values of microvessel density for biochemical recurrence among different PCa populations: A systematic review and meta‐analysis

**DOI:** 10.1002/cam4.5093

**Published:** 2022-08-07

**Authors:** Jinjiang Jiang, Jinze Li, Xingyu Xiong, Shiyu Zhang, Daqing Tan, Lu Yang, Qiang Wei

**Affiliations:** ^1^ Department of Urology, Institute of Urology, West China Hospital Sichuan University Chengdu PR China

**Keywords:** angiogenesis, biochemical recurrence, clinical T stage, microvessel density, prostate cancer

## Abstract

**Background:**

Several studies have explored the relationship between intratumoral microvessel density (MVD) and the risk of postoperative biochemical recurrence (BCR) in prostate cancer (PCa), although the results are contradictory. Therefore, we conducted a meta‐analysis to investigate the effect of MVD on BCR in PCa.

**Method:**

We searched PubMed, MEDLINE, Science Direct/Elsevier, the Cochrane Library, CNKI, and EMBase databases from inception through January 2022, with no year or language restrictions, and used NOS guidelines to evaluate the quality of the 19 eligible studies. The derived hazard ratio (HR) and 95% confidence interval (95%CI) were used to assess each endpoint. Data synthesis was performed with RevMan to assess the prognostic value of MVD in PCa and its heterogeneity, while the publication bias was examined using STATA 16.0.

**Results:**

Our meta‐analysis included 19 articles (4 for T1‐2, 6 for T1‐3, and 9 for T1‐4) on postoperative biochemical recurrence of PCa, among which, 3933 patients were pooled. The predictive ability of intratumoral MVD for different stages of PCa on BCR was T1‐2 (HR, 2.46; 95% CI, 1.08–5.58; *p* = 0.03; *I* ^2^ = 83%), T1‐3 (HR, 2.38, 95% CI, 1.41–4.01; *p* = 0.001; *I* ^2^ = 82%), T1‐4 (HR, 1.61; 95% CI, 1.19–2.19; *p* = 0.002; *I* ^2^ = 61%).The subgroup analyses based on European and immunohistochemical antibody none‐factor VII were consistent with primary one. Sensitivity analysis excluding those studies judged to be at high risk of bias in T1‐2 showed a HR of 2.99[1.70,5.27] (*I* ^2^ = 38%, *p* = 0.0001), demonstrating the robustness of risk estimates of MVD for the assessment of biochemical recurrence.

**Conclusion:**

Microvessel density is a predictor of BCR among patients with PCa, and earlier T stage PCa with a stronger MVD is associated with BCR. Further studies are needed to investigate neoangiogenesis in different T stages of PCa and whether MVD will be of benefit to the EAU‐recommended tool for biochemical recurrence risk assessment.

## Graphical abstract

Microvessel density is a predictor of biochemical recurrence among prostate cancer patients.Moreover,in earlier T stage PCa is, a stronger microvessel density is associated with biochemical recurrence.

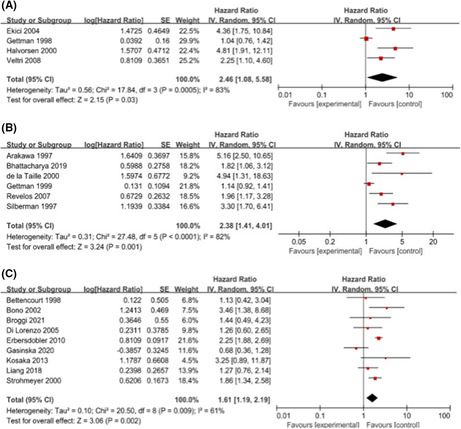

## 1 INTRODUCTION

Prostate cancer (PCa) is the second most commonly diagnosed cancer and the fifth leading cause of cancer‐related deaths among men worldwide, with an estimated 1,414,259 new cancer cases and 375,304 deaths in 2020.^1^ Among newly diagnosed patients with PCa, 90% have clinically localized PCa (T1 and T2) where the primary tumor is confined to the fibrous capsule,^2^ with a tendency to grow slowly and remain asymptomatic or even untreated. As a result, patients with clinically localized PCa tend to undergo less aggressive treatment, such as radical prostatectomy (RP) or radiotherapy treatment, together with active post‐treatment monitoring. However, patients with clinically localized PCa who undergo RP tend to have a lower mortality than those who receive additional treatments.^3^ For postoperative patients with PCa, regular prostate specific antigen (PSA) detection is of great significance to recognize tumor progression.^4^ A previous study suggested that among men with a PSA doubling time (PSADT) < 9 months after the PSA increase, salvage androgen deprivation therapy was significantly associated with a decreased risk of all‐cause and cancer‐specific mortality in the prostatectomy and radiotherapy cohorts.^5^ Additionally, biochemical recurrence (BCR), defined as persisting or rising postoperative PSA(>0.2 ng/mL) on two consecutive measurements, was a common indicator for active surveillance, and Thomas et al. reported that BCR is an independent risk factor for the development of distance metastasis, cancer‐specific mortality, and, to a lesser extent, overall mortality.^6^

Locally advanced PCa, defined as T3 and T4 clinically, commonly invades the surrounding tissue through the capsule, and with the onset of PSA failure after local and systematic treatment, salvage therapy, including second‐generation androgen‐deprived drugs and targeted radiation, is generally considered.^7^ Furthermore, BCR has become a valid surrogate endpoint for PCa‐specific death, which may reduce the follow‐up time in clinical trials.^8^

Overall, the incidence of BCR is affected by many factors, including the preoperative PSA level, surgical margins, Gleason score, obesity and hypertension.^9^, ^10^ The European Association of Urology (EAU) has published criteria for low‐, intermediate‐, and high‐risk groups for BCR of PCa based on preoperative PSA, Gleason score, and clinical stage, and has reported that the BCR risk grouping reached an independent predictor status for metastasis progression (hazard ratio [HR], 3.46; *p* < 0.001) and PCa mortality (HR, 5.12; *p* < 0.001).^11^ This classification can accurately identify patients at low risk in clinical practice, but there is significant overlap among localized patients in the intermediate‐ and high‐risk categories as the criteria do not account for all risk factors.^12^ Thus, more favorable predictors should be introduced to improve the accuracy of predicting BCR of PCa.

Angiogenesis is the basis of tumor growth, infiltration, and metastasis, and previous studies have found that when the tumor size reaches 1–2 mm, tumor growth is limited, and new blood vessels are required to provide long‐term support for tumor growth and dissemination.^13^ Tumor growth beyond the original sufficient blood supply results in local hypoxia and the production and accumulation of numerous HIF‐1a molecules, which activate downstream molecules, including VEGF, FGF, and PDGF‐β to promote angiogenesis, tumor progression, and metastasis.^14^ To observe tumor neoangiogenesis directly, the “hot spot” method, which was first performed by Weidner in breast cancer, is used to count microvessel density in tissue sections. The microvessels are stained for CD31, CD34, or FactorVII, before counting on a microscope, as described previously.^15^ Numerous studies have investigated the correlation between intratumoral microvessel density (MVD) and clinical outcomes in various cancers, including PCa.^16^, ^17^, ^18^, ^19^, ^20^, ^21^, ^22^, ^23^, ^24^, ^25^, ^26^, ^27^, ^28^, ^29^, ^30^, ^31^, ^32^, ^33^, ^34^ Some researchers have found that MVD is associated with postoperative biochemical recurrence,^17^, ^18^ while others have not.^16^, ^22^ This discrepancy may be due to the inclusion of PCa at different stages, where tumor vascular development varies.

In conclusion, the purpose of this study was to explore whether MVD can predict postoperative BCR among patients with PCa and to reveal the extent to which neovascularization is involved in tumor progression in PCa at different stages.

## 2 METHOD

This study was conducted according to the PRISMA guidelines.

### 2.1 Search strategy

Two investigators (Jinze Li and Xingyu Xiong) searched PubMed, MEDLINE, Science Direct/Elsevier, the Cochrane Library, CNKI, and Embase, with no limitations on year, and the final search date was January 25, 2022. The three keywords used were PCa, microvessel density, and prognosis. PCa included “PCa,” “prostate tumor,” “prostate neoplasm,” and “prostate carcinoma”; for prognosis, “Prognos*,” “prognostic factor,” “outcome,” “survival” “PSA recurrence,” “PSA failure,” “predictor,” and “predict” were used; and for microvessel density, “mvd,” “microvessel densit*,” “microvessel count,” “neovascularization,” “microvascular densit*,” “vascularity,” “CD34,” “CD31,” “vwf,” and “vascular density” were used. We also retrieved citations from a manual search.

### 2.2 Criteria for inclusion

To access eligible articles, studies were required to meet certain inclusion and exclusion criteria.

#### 2.2.1 Inclusion criteria

1. Exploring the relationship between MVD and the risk of biochemical recurrence.
2. Patients with PCa had not received neoadjuvant therapy or invasive biopsy before tumor resection.
3. Classification of patients with PCa was based on TNM staging.
4. MVD visualized by CD31, CD34, and Factor VII.
5. MVD located in the primary tumor tissue (not metastases or adjacent normal tissue).
6. Evaluation of outcomes on MVD including BCR.
7. Include cohort studies and randomized controlled studies.

#### 2.2.2 Exclusion criteria

1. Meeting abstracts, reviews, descriptive studies, case reports, commentaries.
2. Sample size <20.
3. Inadequate information on HR calculations and data synthesis.
4. Non‐English language

### 2.3 Data extraction

The information for each study was extracted independently by two investigators (Jinze Li and Xingyu Xiong) with a standardized data form including the following items: first author, publication year, patient source, age, follow‐up, disease stage, specimen acquisition, and results. Among these articles, HR from multivariate analysis was preferred, followed by univariate analysis and Kaplan–Meier survival curve. The primary endpoint was biochemical recurrence, and the secondary endpoint was tumor progression. For studies lacking HR, we calculated the HR using survival curves as described by Tierney.^35^

### 2.4 Risk of bias (RoB) assessment

The RoB in the selected studies was assessed by two authors using the Newcastle‐Ottawa Scale (NOS) tool, which assesses selection, performance, detection, attrition, reporting, and other sources of bias (Supplementary Table S1). The RoB of each study was assessed independently by two authors, and disagreements were resolved by consultation with coauthors; studies that achieved ≥5 on the NOS tool were included, and those with scores >7 were considered high quality.

### 2.5 Statistical analyses

In assessing the BCR for PCa of T1‐2, T1‐3, and T1‐4, the log HR and the standard error calculated from the published HR and confidence interval (CI) were retrieved. A meta‐analysis was conducted for each outcome using a random effect models for the predictive value of MVD. The relative predictive effects are presented as the HR and 95% CI.We planned to perform a subgroup analysis based on (1) ethnicity and (2) immunohistochemical antibodies none‐Factor VII. STATA 16.0 was employed to assess publication bias via a funnel graph and Egger's test. When publication bias existed, we used trim and fill method via STATA 16.0 to examine the stability of the findings. All statistical analyses were performed using Review Manager 5.4 software and STATA 16.0 software. Statistical significance was defined as *p* < 0.05 (two‐sided).

## 3 SYNTHESIS OF EVIDENCE

### 3.1 Study description

**FIGURE 1:**
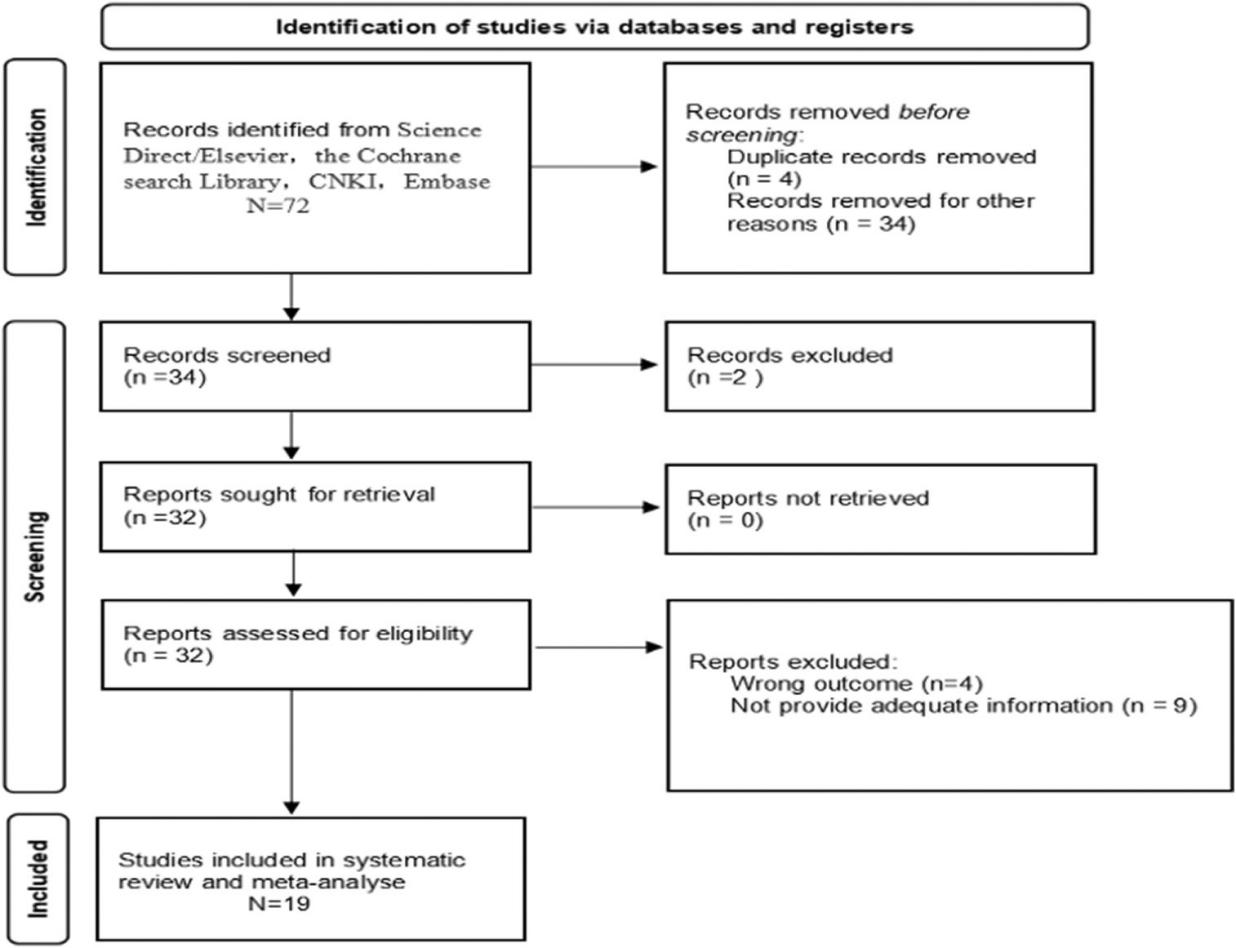
Preferred reporting items for systematic reviews and meta‐analyses flow diagram outlining search strategy and final included studies.

We searched 72 articles, of which 34 were excluded during abstract reading. Thirty‐eight articles were full‐text reviews, of which 19 met our requirements, and each study source was extracted for data synthesis. The reasons for exclusion of articles are presented in Figure 1. To avoid duplication, for more than one publication from the same cohort, we selected studies with more patients and those of higher quality. We displayed the items of the included studies, including the authors, years, origin of population, study design, sample access, sample size, clinical characteristics, median age, median follow‐up, antibody, MVD assessment, endpoint, results, and HR estimated in Table 1.

**TABLE 1:** Characteristic of included studies

First author	Origin of population	Study design	Sample access	Sample size	Clinical characteristic	Median(y) age
Gettman 1998	America	Prospective cohort study	RP	147	T2, GS 6–9	67.5
Halvorsen 2000	Norway	Prospective cohort study	RP	104	T1‐2	NA
Ekici 2004	America	Prospective cohort study	RP	66	T1c,T2a‐b	NA
Veltri 2008	America	Prospective cohort study	RP	105	T2	NA
Silberman 1997	Canada	Prospective cohort study	RP	87	T1‐3, GS 5–7	NA
Arakawa 1997	America	Prospective cohort study	RP	101	T2‐3	63.8
Gettman 1999	America	Prospective cohort study	RP	211	T1‐3	67.3
de la Taille 2000	America	Prospective cohort study	RP	102	T2‐3	62
Revelos 2007	Greece	Prospective cohort study	RP	94	T1‐3	66
Bhattacharya 2019	UK	Prospective cohort study	TURP/TRUbiopsy	204	T1‐3	NA
Bettencourt 1998	America	Prospective cohort study	RP	149	T1‐4	NA
Strohmeyer 2000	Austria	Prospective cohort study	RP	98	T1‐4	NA
Bono 2002	Italy	Prospective cohort study	RP	75	T2‐4, GS 6–10	NA
Di Lorenzo 2005	Italy	Prospective cohort study	RP	72	T2‐4	NA
Erbersdobler 2010	Germany	Prospective cohort study	RP	1521	T2‐4,GS 6–10	63
Kosaka 2013	Japan	Prospective cohort study	RP	167	T1‐4,GS 6–10	66.6
Liang 2018	China	Prospective cohort study	RP	232	T2‐4, GS 6–10	66
Gasinska 2020	Poland	Prospective cohort study	RP	130	T1‐4,GS 6–10	62.8
Broggi 2021	Italy	Prospective cohort study	RP	268	T2‐4,GS 6–10	NA

First author	Median(y) follow‐up	Antibody	MVD assessment	Endpoint	Results	HR estimated
Gettman 1998	6.6	Factor VII	OMVD	BCR + CP	Negative	Multivariate analysis
Halvorsen 2000	4.6	Factor VII	MVD	BCR + CP	Positive	Multivariate analysis
Ekici 2004	8.6	CD34	MVD	BCR	Positive	Univariate analysis
Veltri 2008	17.3	CD31	maxMVD	BCR	Positive	Multivariate analysis
Silberman 1997	8.2	CD31	MVD	BCR	Positive	Multivariate analysis
Arakawa 1997	6.8	CD34/Factor VII	MVD	BCR	Positive	Kaplan–meier survival curves
Gettman 1999	7.1	Factor VII	OMVD	BCR	Negative	Multivariate analysis
de la Taille 2000	3.6	CD34/CD31	MVD	BCR	Positive/Negative	Kaplan–meier survival curves
Revelos 2007	2.3	CD34	MVD	BCR	Positive	Multivariate analysis
Bhattacharya 2019	7.08	CD34	MVD	BCR	Positive	Kaplan–meier survival curves
Bettencourt 1998	6.6	CD34	MVD	BCR	Positive	Kaplan–meier survival curves
Strohmeyer 2000	3.5	Factor VII	MVD	BCR + CP	Positive	Multivariate analysis
Bono 2002	7	CD34	MVD	BCR	Positive	Kaplan–meier survival curves
Di Lorenzo 2005	6	CD31	MVD	BCR	Negative	Multivariate analysis
Erbersdobler 2010	38.9	CD31	MVD	BCR	Positive	Kaplan–meier survival curves
Kosaka 2013	4.9	CD34	MVD	BCR	Positive	Kaplan–meier survival curves
Liang 2018	NA	CD31	MVD	BCR	Negative	Multivariate analysis
Gasinska 2020	78.5	CD34	MVD	BCR	Negative	Univariate analysis
Broggi 2021	NA	CD31	MVD	BCR	Positive	Kaplan–meier survival curves

Abbreviations: BCR, biochemical reccurrence; CP, clinical progression; MVD, microvessel density; NA, not assessed; OMVD, optimized microvessel density; RP, radical prostatectomy; TURP, transurethral resection of prostate.

### 3.2 Biochemical recurrence

**FIGURE 2:**
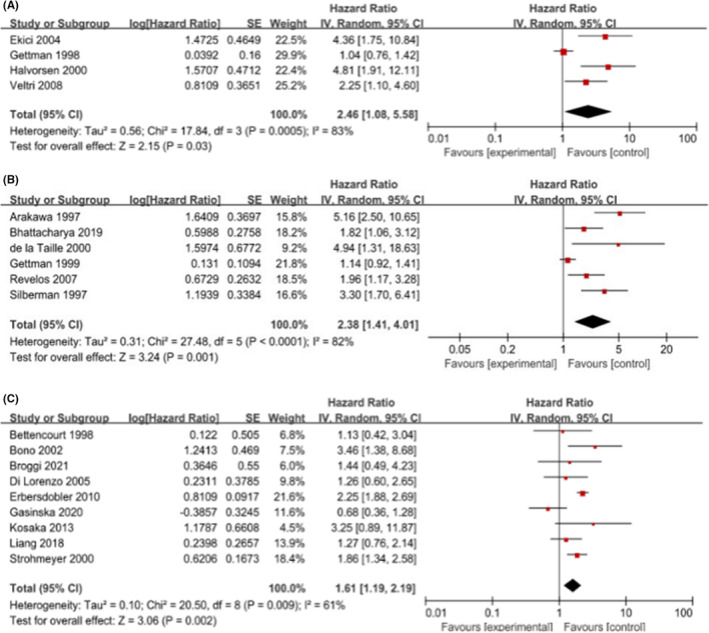
Fig 2‐Forrest plot assessing the risk of biochemical recurrence for (A) stage T1‐2 (B) stage T1‐3 (C) stage T1‐4 following surgery for PCa. CI, confidence interval; IV, inverse variance; SE, standard error.

We performed statistical calculations among the 19 eligible studies associated with biochemical recurrence, including four studies for T1‐2 PCa, six for T1‐3 PCa, and nine for T1‐4 PCa. Individually, the pooled HR and its 95% CI for T1‐2 were 2.46 (1.08, 5.58; *p* = 0.0005), for T1‐3 were 2.38 (1.41, 4.01; *p* = 0.001), and the pooled HR (95% CI) for T1‐4 was 1.61 (1.19, 2.19; *p* = 0.002). The results are shown in Figure 2. Overall, a higher MVD was associated with PSA failure.

**FIGURE 3:**
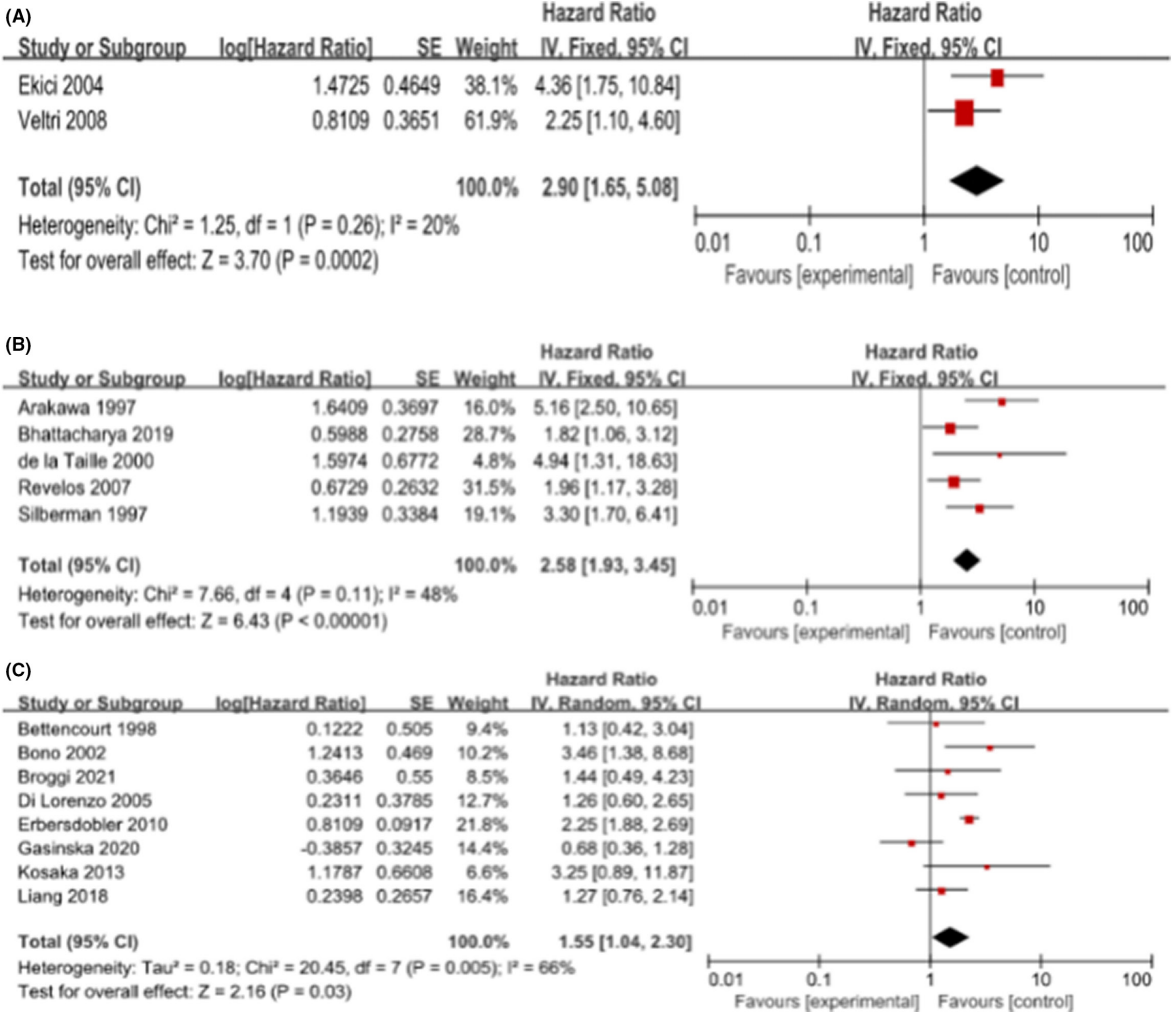
Forrest plot assessing the risk of biochemical recurrence for subgroups (A) stage T1‐2 (B) stage T1‐3 (C) stage T1‐4 with MVD expression in tumor tissue by none‐Factor VII IHC. CI, confidence interval; IV, inverse variance; SE, standard error.

**FIGURE 4:**
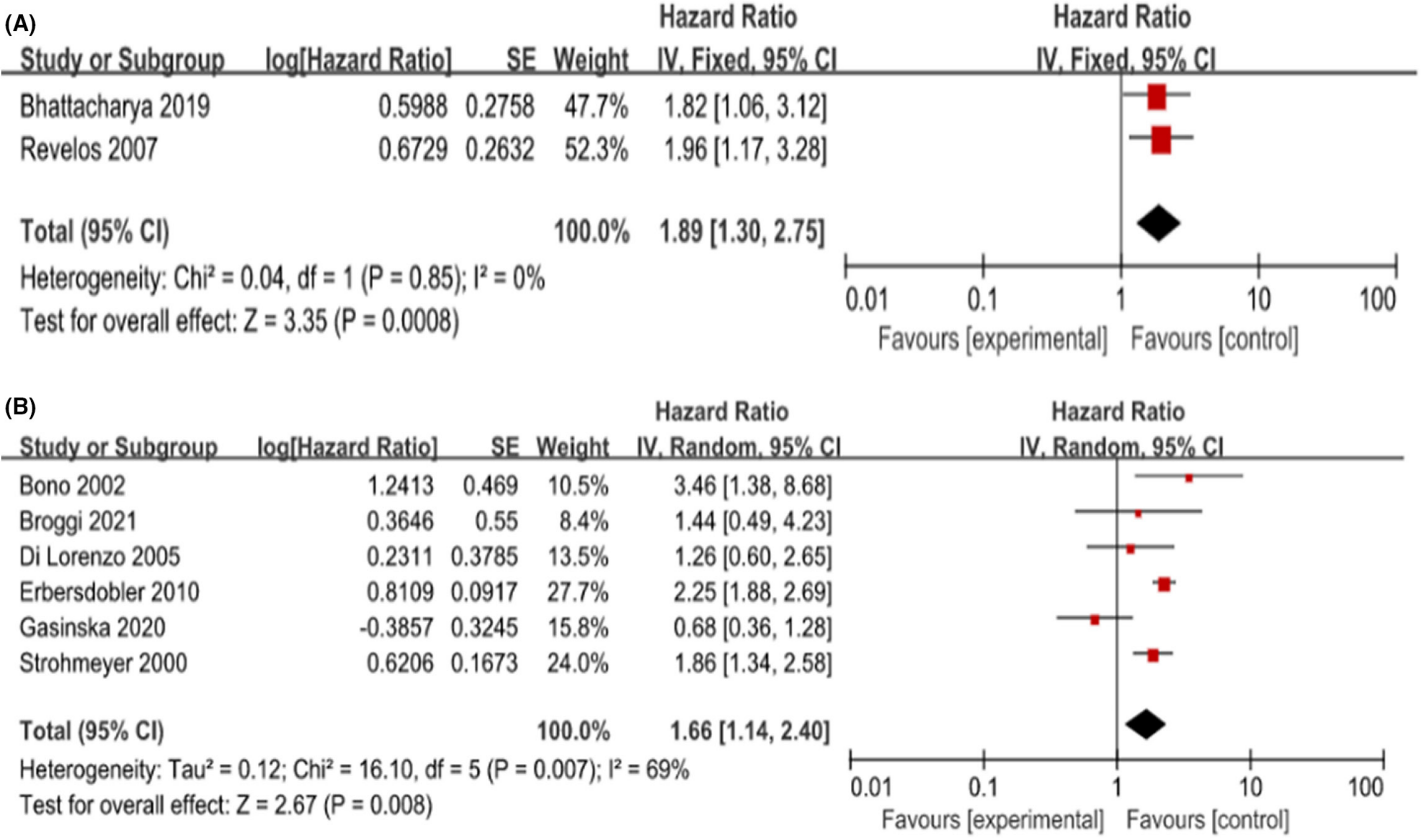
Forrest plot assessing the risk of biochemical recurrence for European subgroups (A) T1‐3 PCa(B) T1‐4 PCa. CI, confidence interval; IV, inverse variance; SE, standard error.

We performed subgroup analysis with the following items: the patients' origin (European) and the antibody used to detect MVD (none‐Factor VII) in immunohistochemical staining. We aggregated the studies from three groups (T1‐2, T1‐3, T1‐4) based on angiogenic markers (none‐Factor VII) to predict the BCR within HRs. As shown in Figure 3, the pooled HR was 2.90 (1.65, 5.08) for T1‐2, 2.58 (1.93, 3.45) for T1‐3, and 1.55 (1.04, 2.30) for T1‐4. These results were consistent with the results of the meta‐analysis above. Furthermore, we found that the pooled HRs for European were 1.89 (1.30, 2.75; *I* ^2^ = 0%, *p* = 0.0008) and 1.66 (1.14, 2.40; *I* ^2^ = 69%, *p* = 0.008) in T1‐3 and T1‐4, respectively. As shown in Figure 4, among the European population in T1‐3 and T1‐4, higher MVD increased the risk of postoperative BCR in patients. In T1‐2, there was only one study from Europe; to avoid causing a significant deviation to the overall analysis, we decided to remove its outcome. Unfortunately, we did not conduct a subgroup analysis for Asian and American due to a lack of adequate data dealing with MVD.

### 3.3 Publication bias

**FIGURE 5:**
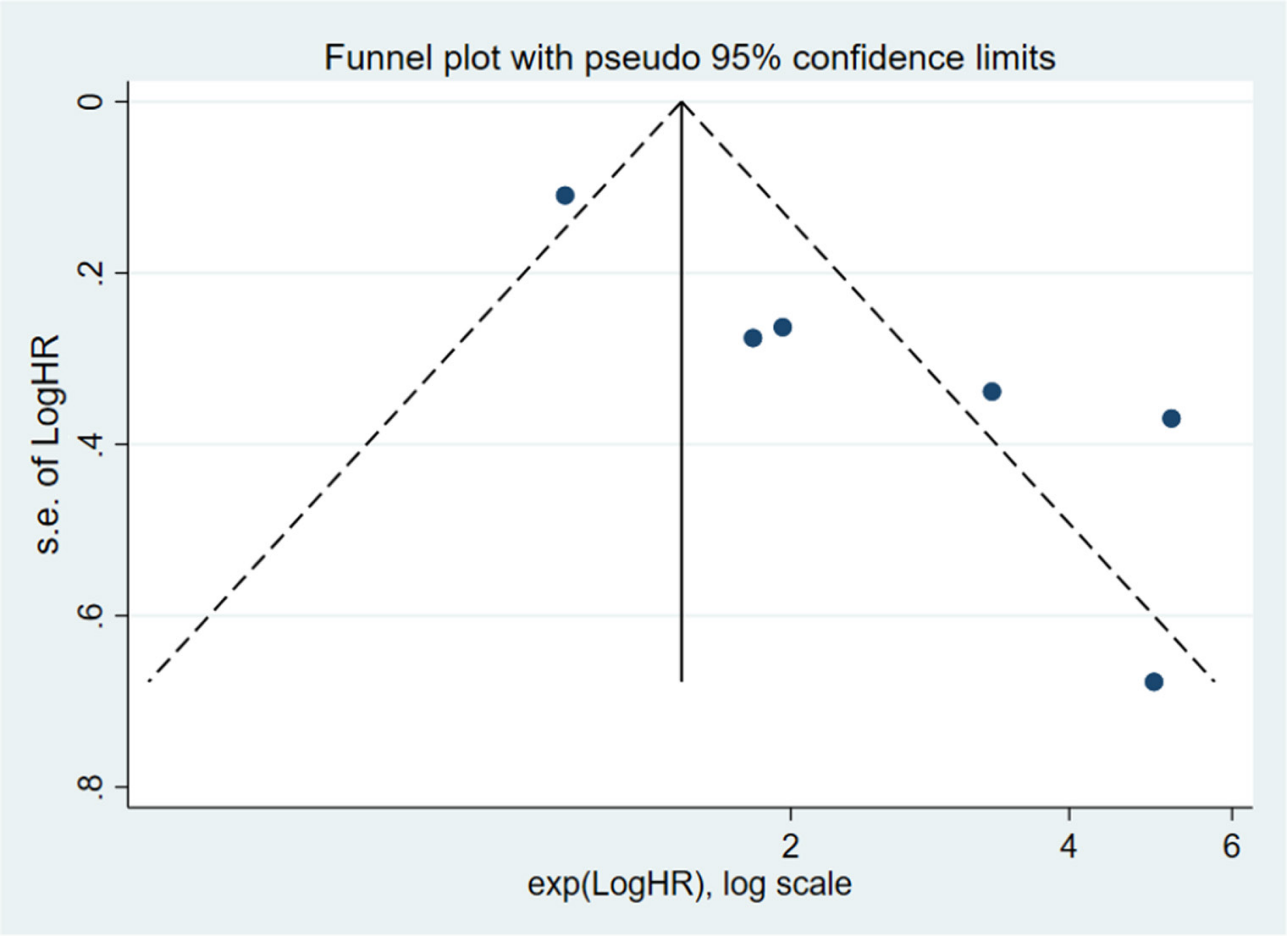
Funnel plot showing: Log hazard ratios of biochemical recurrence for stage T1‐3 following surgery for PCa.

**FIGURE 6:**
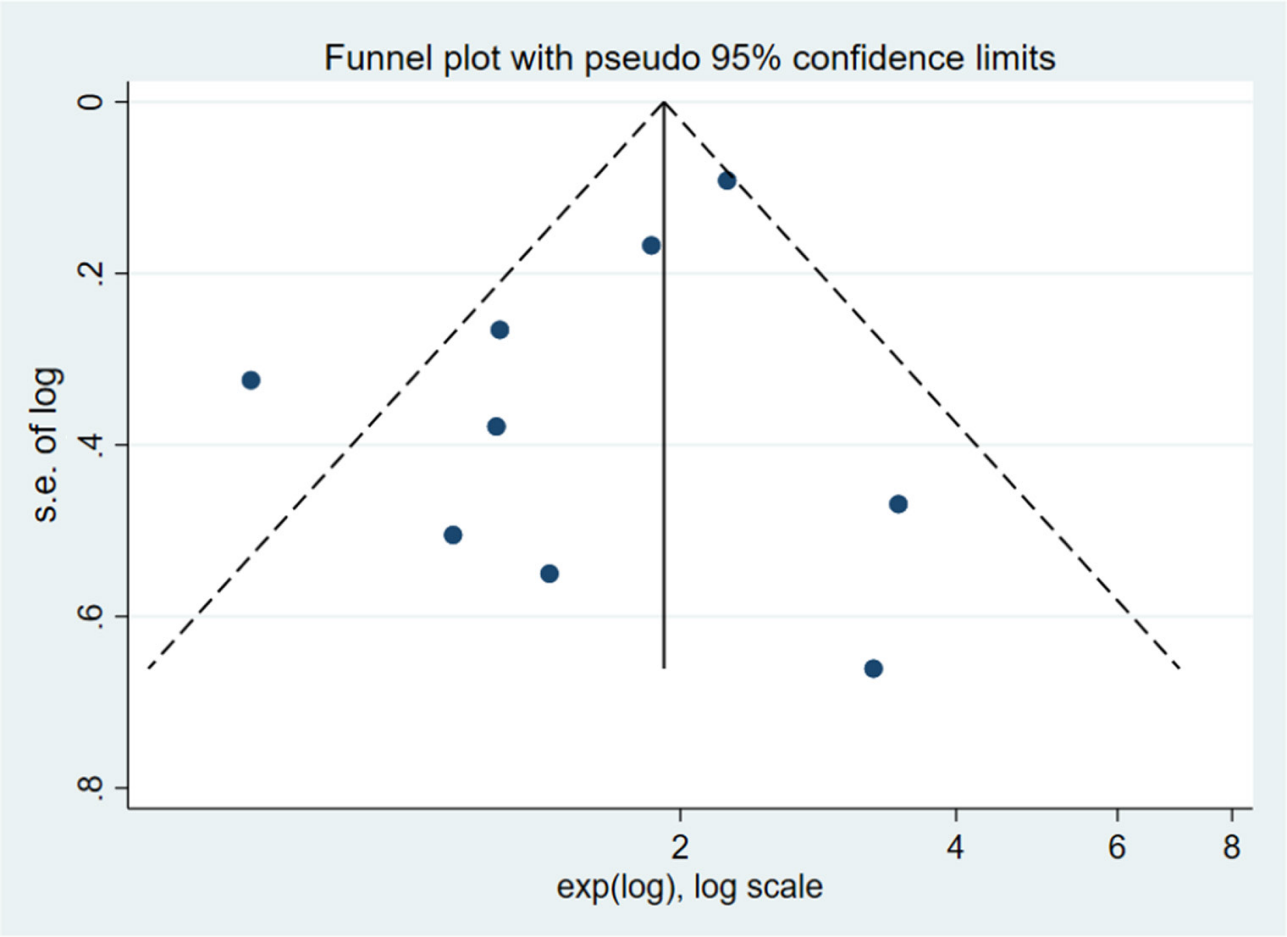
Funnel plot showing: Log hazard ratios of biochemical recurrence for stage T1‐4 following surgery for PCa.

Publication bias was assessed using funnel plots and Egger's test with STATA 16.0. However, total number of articles of T1‐2 PCa was 4, there was a risk of overestimating the validity of the results of pubication bias in T1‐2 PCa. We only performed publication bias on T1‐3 and T1‐4. The funnel plots revealing evidence of potential publication bias are displayed in Figures 5 and 6. Moreover, the p‐values of the Egger's test for each outcome were 0.007 (T1‐3), and 0.136 (T1‐4), respectively. No significant publication bias was found among the included articles for T1‐4. However, there was publication bias among the articles for T1‐3 PCa. We further evaluated the number of missing studies in a meta‐analysis and recalculated the pooled risk estimate with these studies using the trim and fill method. The filled funnel plot is shown in Figure S5 and pooled risk estimate for the stage T1‐3 PCa group was 1.293 (1.100–1.519; *p* = 0.002), which was consistent with the primary one, indicating that publication bias in T1‐3 did not generally change results.

### 3.4 Sensitivity analysis

Overall, a sensitivity analysis was conducted, in which one study was removed at a time, and the results are presented in Figures S1–S3 in the Supplementary Appendix. The distribution of dots indicates that our results were stable. As shown in Figure S4, a further sensitivity analysis was conducted by excluding two studies (Gettman++ 1998 and Ekici 2004), which were judged to be at relatively high RoB (NOS score = 6), the results of which confirmed the substantial risk of BCR with a HR of 2.99 (1.70, 5.27; *I* ^2^ = 38%, p = 0.0001) in the T1‐2 group.

## 4 DISCUSSION

Until now, predictors related to BCR can be roughly divided into two categories: tumor biology including the pathological Gleason score and clinical variables including preoperative PSA level, tumor stage, seminal vesicle invasion, lymph node, and surgical margin status, and are widely used as risk factors for BCR, most of which are incorporated through the nomogram. Raisa et al. reported that their BCR nomogram, which included easily applicable variables, could be used to predict BCR within 12 and 24 months of RP in European patients who received RP.^36^ The overall accuracy values were 81% and 81.1%. In most cases, the nomogram focuses on clinical characteristics other than tumor biology. Therefore, to better establish a prognostic tool, we investigated whether risk factors from the perspective of tumor biology can predict the likelihood of further progression.

We performed a meta‐analysis to explore the ability of MVD to predict the risk of BCR in different stages of PCa. The results of the meta‐analysis demonstrate that, in general, MVD in cancer tissues is associated with postoperative BCR of PCa, but the value of risk prediction differs when considering patients at different clinical stages. For PCa in the T1‐4 population, MVD was associated with poor prognosis, with an HR of 1.61 (1.19–2.19). While only clinically localized prostate was included, the HR for BCR was 2.46 (1.08, 5.58). Interestingly, the risk of PSA recurrence changed when patients were included in different clinical stages. As more higher‐stage patients are included, the HR for predicting BCR in PCa decreases; however, patients diagnosed with pathological stage T3‐4 are prone to undergo more aggressive treatment, such as chemical therapy and anti‐androgen therapy (ADT), after surgery. In contrast, most of the T1‐2 patient population merely undergo surgical resection or radiotherapy and rarely receive additional treatment after surgery, resulting in the patients with PCa in the T1‐2 population being at a higher risk of BCR.

However, from the perspective of tumor progression, angiogenesis is more intensive in early stage PCa confined to the fibrous capsule than in patients with locally advanced PCa, in which the cancer has breached the fibrous capsule and invaded surrounding tissues. The process of tumor vascularization is vital for the early stage of tumor growth and remains vulnerable to inhibitors. Weidner found that PCa tumor cells rarely enter the blood circulation and undergo micrometastasis before the primary tumor is fully vascularized, and tumor progression and metastasis require tumor cells to enter the bloodstream.^37^ Only when cancer cells that survive in the bloodstream are captured by target organs and proliferate and develop in target organs can tumor metastasis occur. Mareel reported that the number of cancer cells with all of the above abilities does not exceed 1/1000.^38^ During tumor progression, a mature vascular network is a prerequisite for tumor growth, providing nutrients and pathways for metastasis, during which, various angiogenesis molecules are upregulated and work through different signaling pathways. Moreover, Elżbieta Łuczyńska observed that the expression of VEGF in pTNM3 and pTNM4 is significantly higher than that in pTNM1.^39^ The differential expression deeply affects the correlation between MVD and tumor progression. Thus, the results of our meta‐analysis implies that the MVD of PCa from the T1‐2 population predicts a higher risk of BCR than that from the T1‐4 population.

Bevacizumab is a monoclonal drug targeting VEGF‐A, which is the main regulator of angiogenesis. Theoretically, bevacizumab can bind to VEGF‐A and interfere with the combination of VEGF‐A and its receptors, blocking the process of tumor angiogenesis and consequently delaying or even inhibiting tumor growth, invasion, metastasis, and other malignant behaviors. Bevacizumab has achieved good performance in the treatment of non‐small cell lung cancer, metastatic colorectal cancer, and breast cancer.^40^ Weidner claimed that vascularization plays an important role in the progression of mCRPC.^37^ In recent years, many clinical trials have explored the feasibility of angiogenesis‐targeted therapy (ATT) in patients with mCRPC.^41^, ^42^, ^43^ In a randomized, double‐blind, controlled clinical trial of docetaxel and prednisone chemotherapy regimen (DP) combined with bevacizumab in patients with mCRPC, the addition of bevacizumab to docetaxel and prednisone did not improve the overall survival (OS) in men with mCRPC and was associated with greater toxicity.^41^ Moreover, the mTOR gene was found to be a regulator in the VEGF pathway and engaged in the process of HIF‐a improvement. Previous studies have reported that mTOR plays an important role in bevacizumab resistance through mTOR upregulation in HIF‐a.^42^ A clinical trial of docetaxel and bevacizumab combined with the mTOR inhibitor everolimus was conducted; however, the outcome showed that this regimen did not effectively improve the OS of patients.^42^ Moreover, it has been reported that thalidomide inhibits the secretion of VEGF and FGF by blocking the PDGFR‐ β signaling pathway, thereby impairing epithelial cellular migration and adhesion abilities and accordingly exerting an antiangiogenic effect.^44^ Clinical trials of thalidomide combined with bevacizumab indicate that the regimen can effectively prolong OS, but its severe toxicity cannot be tolerated by patients.^44^ To date, it seems that little progression has been achieved in anti‐angiogenesis therapy for mCRPC in clinical trials. Because mCRPC is an end‐stage PCa, angiogenesis molecule production and their interactions are complicated, which likely contradicts neovascularization in the early stage of PCa. Higher MVD predicts a risk of BCR among patients from T1‐4, but the HR decreases compared to those from T1‐2. Overall, according to the results of the clinical trials above and our meta‐analysis, we assume that neovascularization was gradually attenuated.

Unlike the poor outcome of mCRPC treated with bevacizumab, bevacizumab achieved satisfactory results in clinically localized PCa. In a phase II clinical trial of neoadjuvant docetaxel plus bevacizumab in high‐risk localized patients with PCa, 29% of patients achieved a >50% reduction in tumor volume. It is encouraging that bevacizumab combined with neoadjuvant chemotherapy can reduce the tumor volume and decrease the difficulty of cancer resection.^44^ Another clinical trial was performed on patients with clinically localized PCa who were administered bevacizumab after local treatment. Consequently, bevacizumab was proven to prolong the time to PSA doubling (from 4.7 to 6.5 months) and the median time to PSA progression after surgery.^45^ Thus, the results of the clinical trials on bevacizumab showing that patients with early‐stage PCa are prone to benefit from angiogenesis targeted therapy, indicating that angiogenesis in early stage PCa is fragile and susceptible to anti‐vascular treatment with bevacizumab. Furthermore, the predictive value of MVD for PSA failure in the T1‐2 PCa population seems stronger than that in the T1‐4 population. Considering the outcome of clinical research and pathological MVD, we propose that angiogenesis is essential in the early stages of tumorigenesis and that angiogenesis‐targeted therapy with VEGF‐A can benefit patients in the early stages.

The discrepancy in the predictive value and efficacy of anti‐angiogenesis therapy in different stages of PCa suggests that tumor vascular development and response to antivascular therapy are highly associated with tumor progression. In addition to VEGF, PDGF, FGF, and other typical angiogenesis molecules, Boddy et al. claimed that androgens may regulate VEGF levels by activating HIF in androgen‐sensitive tumors.^46^ Compared to the androgen‐sensitive growth of prostate tumors at the early stage, mCRPC is considered to have entered an androgen‐resistant growth phase; therefore, these findings suggest that the interaction between androgens and vascular growth factors during angiogenesis may be useful to study angiogenesis and tumor progression of PCa at different stages.

Currently, with numerous drugs targeting angiogenesis molecules, whether an angiogenesis‐targeted regimen can be a promising choice for PCa requires further exploration among different stages of PCa. The underlying mechanism may involve a complex interaction of angiogenesis‐related molecules and androgens in PCa at different clinical stages, resulting in different predictive values of MVD and discrepancies in the efficacy of angiogenesis‐targeted therapy.

The conventional BCR stratification tool proposed by the EAU was based on the GS score, clinical stage, and preoperative PSA. Cooperberg^12^ reported that the estimated risk of BCR for patients with preoperative PSA levels of 10.1–20 ng/mL, 20.1–30 ng/mL, and > 30 ng/mL were as follows: HR, 2.51, 95% CI (1.75–3.59); HR, 3.39, 95% CI (1.82–6.32); and HR, 4.72, 95% CI (2.49–8.99), respectively. For patients with GS scores of 1–3/4–5, 4–5/1–5, the corresponding HRs were 1.55, 95% CI (1.10–2.18) and 3.26, 95% CI (2.23–4.76). Moreover, for patients with clinical stage cT3a vs cT1‐cT2, the risk of BCR was HR, 1.51 (95% CI, 0.67–3.42). As shown above, PSA and Gleason score were the greatest predictors of BCR. In contrast, the HR of higher MVD in sections predicting BCR in T1–4 was 1.61, 95% CI (1.19–2.19). Although MVD is a favorable predictor for BCR at T1‐4, its potential is lacking in clinical practice. However, for patients with T1‐2 and T1‐3 disease, the risk of BCR was 2.46, 95% CI (1.08, 5.58) and 2.38, 95% CI(1.41, 4.01), respectively. Combined with the results of Cooperberg, higher MVD counts in pathological tissue could strengthen the use of T1‐3 clinical stage for BCR prediction among patients with PC, which could complement the BCR risk stratification tool in EAU. However, this requires further validation in a large and high‐quality clinical cohort study.

## 5 LIMITATIONS

We failed to perform publication on T1‐2 PCa. Only 4 studies on clinically localized PCa (T1‐2) were included, for publication bias, there is a risk of overestimating the validity of the results of publication bias.^47^ For the sake of better understand the results from T1‐2, we optimized our retrieval strategy to reconduct the research but no more studies were included. Moreover, sensitivity analysis excluding these two studies (Gettman, 1998 and Ekici, 2004) judged to be at relative high risk of bias confirmed this substantial risk of BCR with an HR of 2.99 (1.70, 5.27; *I* ^2^ = 0.0001) among T1‐2 group. Overall, after confirming no extra missing study, performing sensitivity analysis, the original results were considered to be convincing.

We were unable to conclude a quantitative relationship between MVD and PSA relapse time because the median MVD and follow‐up duration were highly heterogeneous among the included studies.

## 6 CONCLUSION

Microvessel density can predict postoperative BCR in different stages of PCa, and as tumor stage increases, the risk of BCR decreases. Whether MVD prediction can supplement existing EAU BCR risk stratification tools requires further research.

### AUTHOR CONTRIBUTION

Jinjiang Jiang contributed to choose research directions. Jinze Li,Xingyu Xiong, Lu Yang were responsible for literature retrieval. Jinze Li, Qiang Wei and Xingyu Xiong contributed to literature inclusion. Shiyu Zhang and Daqing Tan assessed the level of evidence. Jinjiang Jiang and Jinze Li finished the manuscript. All authors read and approved the publication of final manuscript.

### CONFLICT OF INTEREST

The authors declare that there are no conflicts of interest.

## DATA AVAILABLE STATEMENT

The data that support the findings of this study are available from the corresponding author upon reasonable request.

## ETHICS STATEMENT

Not applicable.

## Supporting information




Figure S1
Figure S2
Figure S3
Figure S4
Figure S5
