# Supplementary material for: Different predictive values of microvessel density for biochemical recurrence among different PCa populations: A systematic review and meta‐analysis
Source: Cancer Med. 2022 Aug 7;12(3):2166–78. doi: 10.1002/cam4.5093 (PMC9939166; doi:10.1002/cam4.5093)
Supplement: Supplementary file 1 — Figure S1 Figure S2 Figure S3 Figure S4 Figure S5 [file CAM4-12-2166-s001.docx]

Supplementary Figure 1: Sensitivity analysis for stage T1-2 following surgery for PCa. CI = confidence interval.


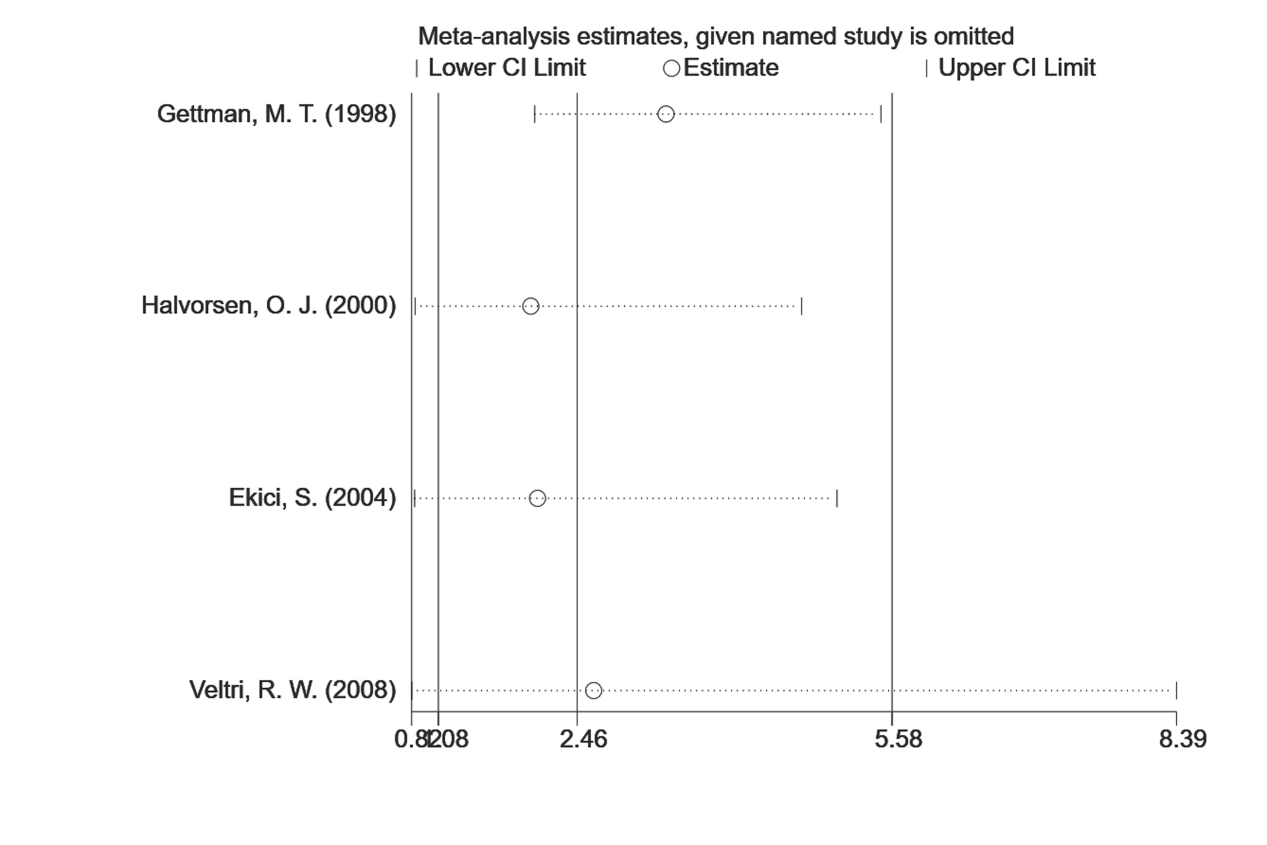


Supplementary Figure 2: Sensitivity analysis for stage T1-3 following surgery for PCa. CI = confidence interval.


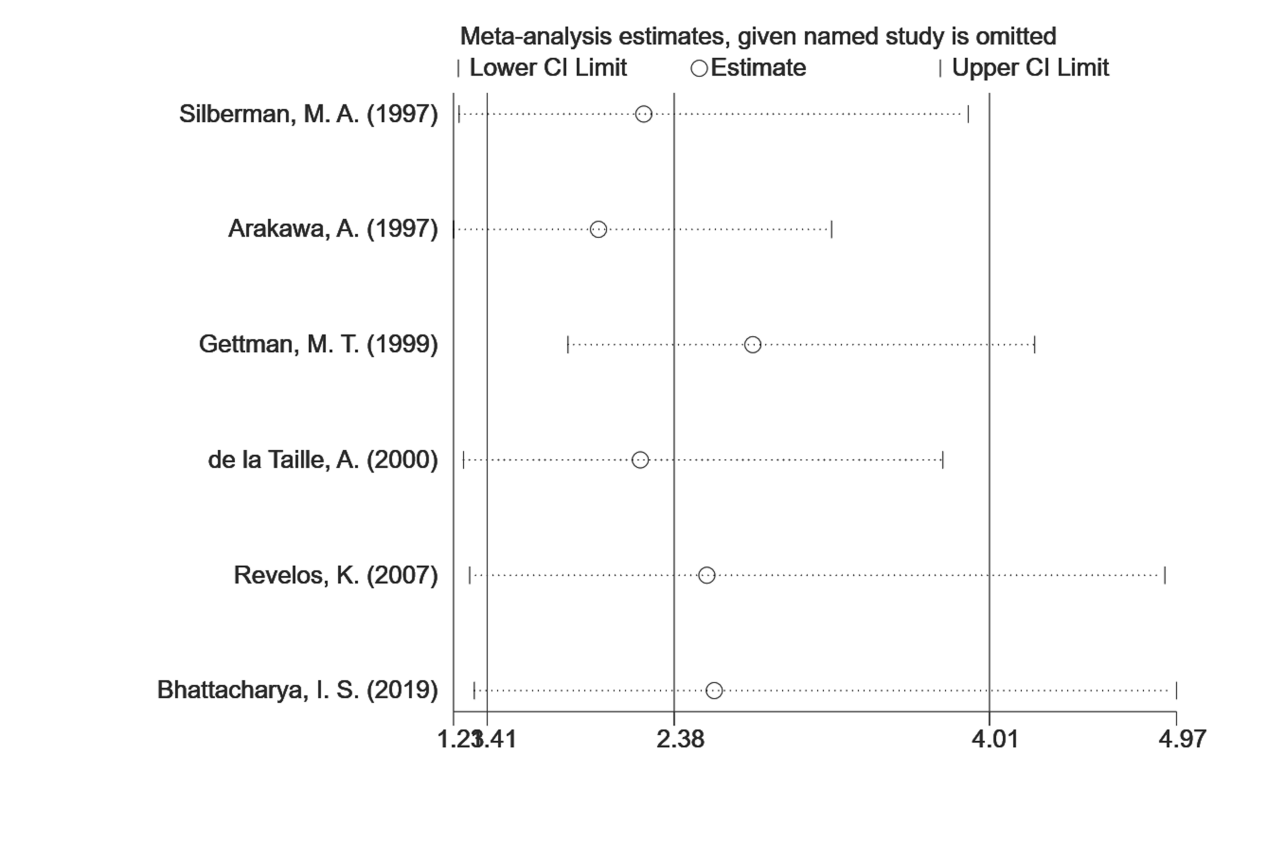


Supplementary Figure 3: Sensitivity analysis for stage T1-4 following surgery for PCa. CI = confidence interval.


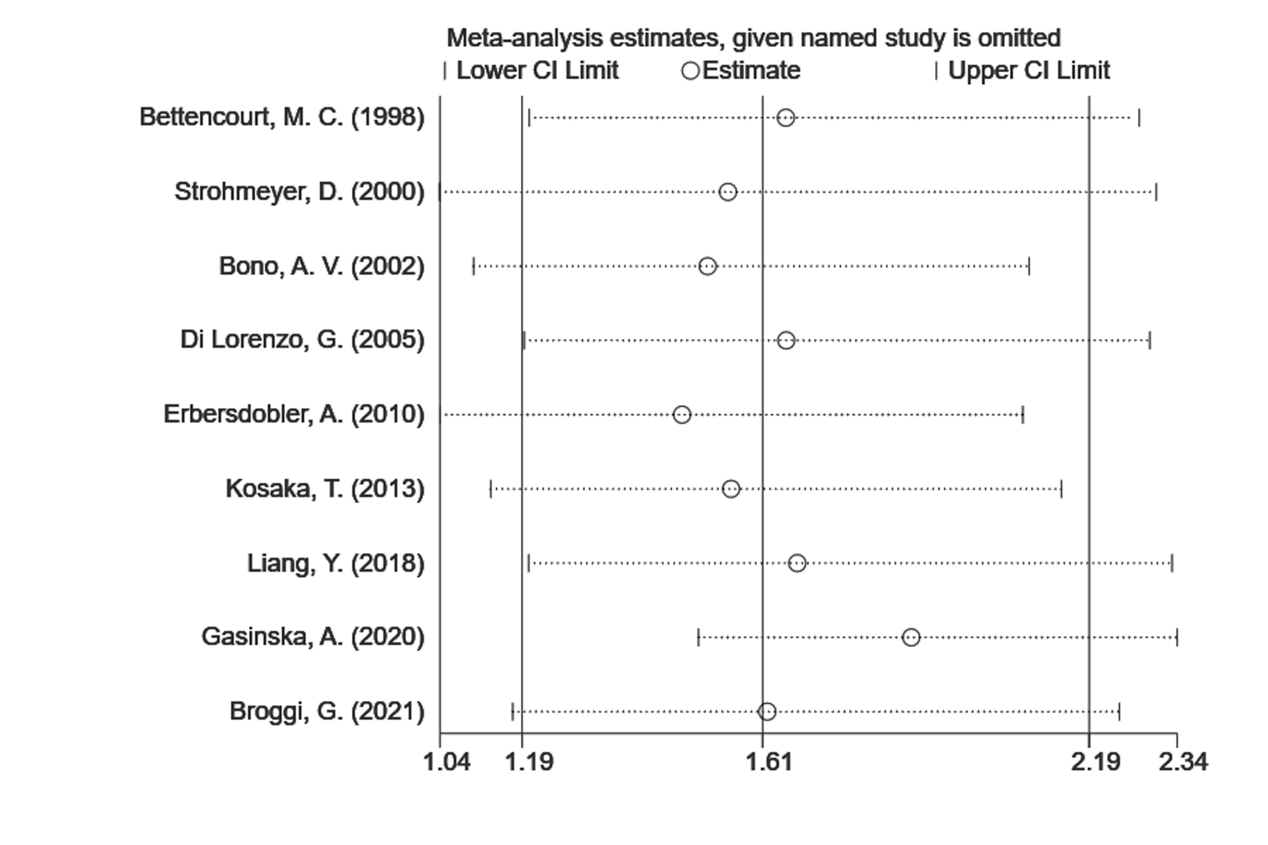


Supplementary Figure 4: Sensitivity analysis assessing the risk of biochemical recurrence for stage T1-2 following surgery for PCa. CI = confidence interval; IV = inverse variance; SE = standard error.


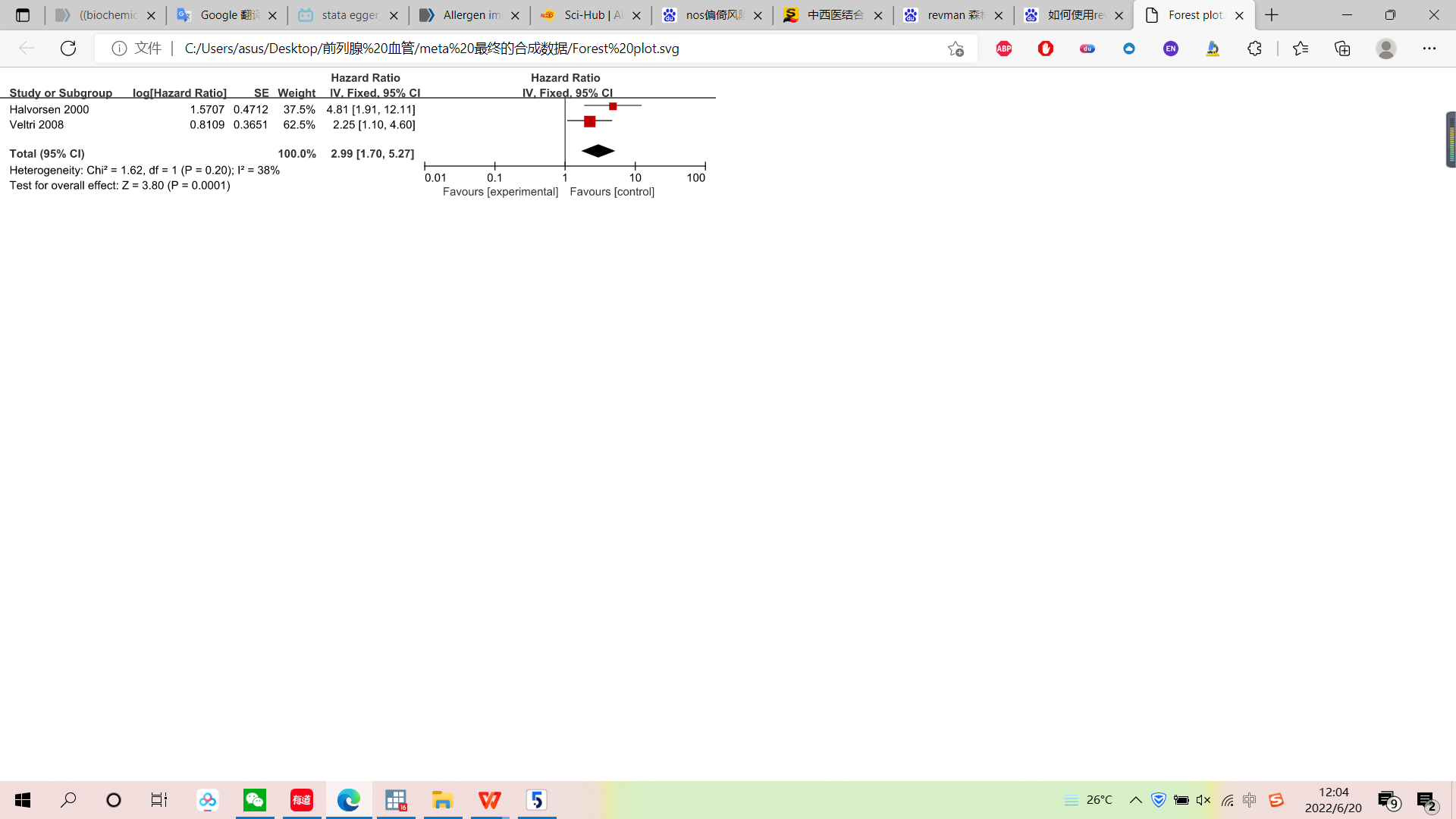


Supplementary Figure 5: Trim and fill procedure stage T1-3 publication


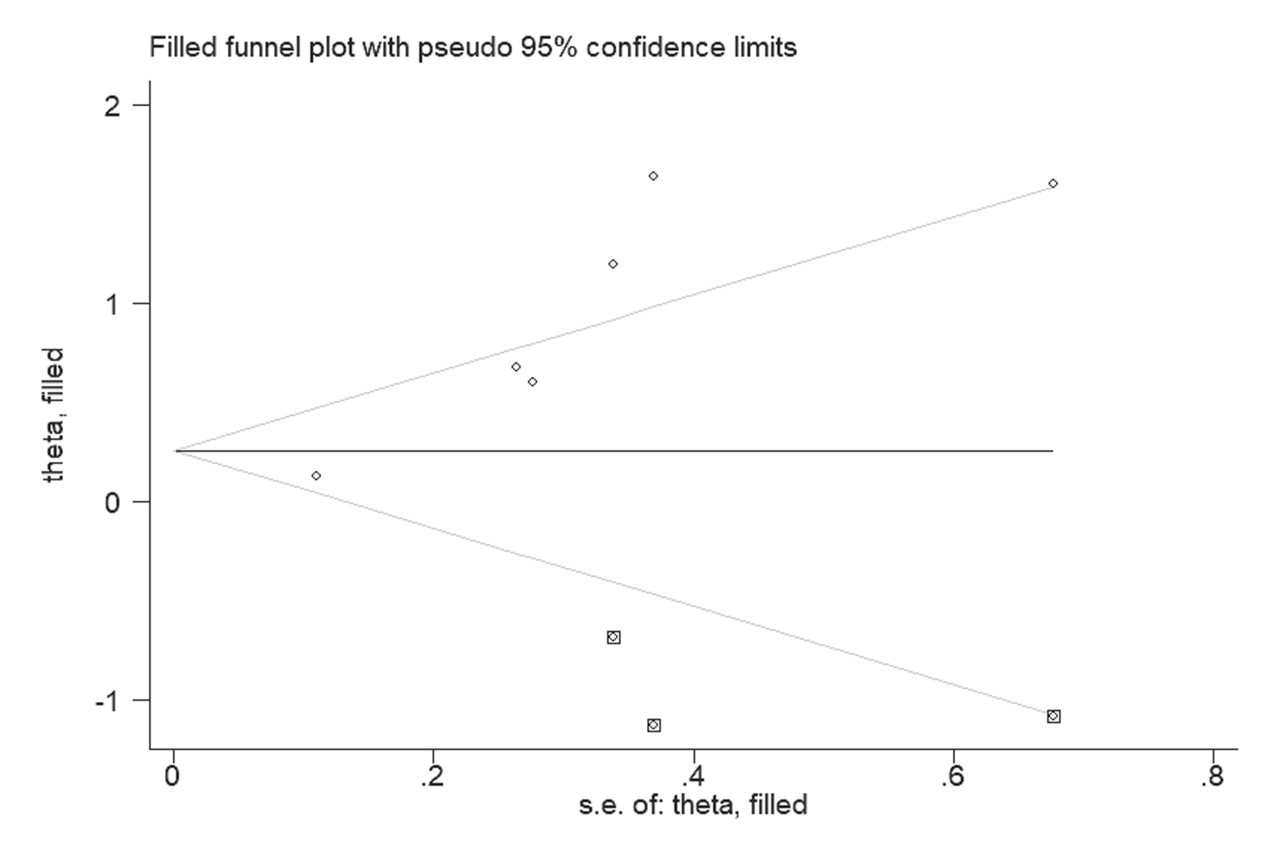


| **Author(publish year)** | **Selection** | | | | **Comparability** | **Outcome** | | | **total** |
| --- | --- | --- | --- | --- | --- | --- | --- | --- | --- |
|  | **exposed cohort** | **non exposed cohort** | **Ascertainment of exposure** | **outcome** | **Comparability** | **Assessment of outcome** | **follow-up** | **Adequacy of**  **follow up of cohorts** |  |
| Gettman（1998） | 0 | 0 | 1 | 1 | 1 | 1 | 1 | 1 | 6 |
| Halvorsen(2000) | 1 | 1 | 1 | 1 | 2 | 1 | 1 | 1 | 9 |
| Ekici(2004) | 0 | 0 | 1 | 1 | 1 | 1 | 1 | 1 | 6 |
| Veltri(2008) | 1 | 1 | 1 | 1 | 2 | 1 | 1 | 1 | 9 |
| Silberman(1997) | 1 | 1 | 1 | 1 | 2 | 1 | 1 | 1 | 9 |
| Arakawa(1997) | 1 | 1 | 1 | 1 | 1 | 1 | 1 | 1 | 8 |
| Gettman(1999) | 1 | 1 | 1 | 1 | 2 | 0 | 1 | 1 | 8 |
| de la Taille(2000) | 1 | 1 | 1 | 1 | 1 | 1 | 1 | 1 | 8 |
| Revelos(2007) | 1 | 1 | 1 | 1 | 2 | 1 | 1 | 1 | 9 |
| Bhattacharya(2019) | 1 | 1 | 1 | 1 | 2 | 1 | 1 | 1 | 9 |
| Bettencourt(1998) | 1 | 1 | 1 | 1 | 1 | 1 | 1 | 1 | 8 |
| Strohmeyer(2000) | 1 | 1 | 1 | 1 | 2 | 0 | 1 | 1 | 8 |
| Bono(2002) | 1 | 1 | 1 | 1 | 1 | 1 | 1 | 1 | 8 |
| Di Lorenzo(2005) | 1 | 1 | 1 | 1 | 2 | 1 | 1 | 1 | 9 |
| Erbersdobler(2010) | 1 | 1 | 1 | 1 | 2 | 1 | 1 | 1 | 9 |
| Kosaka(2013) | 1 | 1 | 1 | 1 | 1 | 1 | 1 | 1 | 8 |
| Liang(2018) | 1 | 1 | 1 | 1 | 2 | 0 | 1 | 1 | 8 |
| Gasinska(2020) | 1 | 1 | 1 | 1 | 1 | 1 | 1 | 1 | 8 |
| Broggi(2021) | 1 | 1 | 1 | 1 | 1 | 1 | 1 | 1 | 8 |

Supplementary Table1. NOS tool evaluate risk of bias on included study
